# Aerodigestoscopy (ADS): The Feasibility, Safety, and Comfort of a Comprehensive Procedure for the Evaluation of Physiological Disorders of the Aerodigestive Tract

**DOI:** 10.3390/jcm13247578

**Published:** 2024-12-13

**Authors:** Robert J. Arnold, Nina Bausek, Christopher S. Gaskill, Lawrence F. Johnson, Sigfredo Aldarondo, Cody Aull, Malik Midani, Tarek Midani, Ramiz Midani, Ashton S. Brown, Alana Wallace

**Affiliations:** 1 Southeastern Biocommunication Associates, LLC, Birmingham, AL 35216, USA; chris.gaskill@gmail.com (C.S.G.); mikex535@gmail.com (M.M.); trkmid@gmail.com (T.M.); ramiz.r.star37@gmail.com (R.M.); ashtonsbrown@outlook.com (A.S.B.); alanamarie3320@gmail.com (A.W.); 2 Department of Cardiovascular Medicine, Mayo Clinic, Rochester, MN 55905, USA; bausek.nina@mayo.edu; 3 Division of Gastroenterology and Hepatology, University of Alabama at Birmingham, Birmingham, AL 35233, USA; lfjmd@bellsouth.net; 4 Pulmonary Rehabilitation Program, AdventHealth Orlando, Orlando, FL 32825, USA; saldarondo@gmail.com; 5 Otolaryngologist—Head & Neck Surgeon, National Breathe Free Sinus & Allergy Centers, West Palm Beach, FL 33401, USA; codyaull@yahoo.com

**Keywords:** aerodigestive disorders, FEES, stroboscopy, endoscopy, aerodigestive tract, swallowing, dysphagia, dysphonia, aerodigestive physiology, gastroesophageal exam

## Abstract

**Background:** Limited access to the comprehensive assessment of disorders of the upper aerodigestive tract is a barrier to care in rural health facilities. Assessment of the full aerodigestive tract requires a variety of specialists. The inability to access the necessary specialists can result in misdiagnosis, underdiagnosis, and increased associated mortality. Currently, no single differential diagnostic exam comprehensively assesses all functions of the upper aerodigestive tract to bridge this gap. The purpose of this study is to investigate the feasibility and safety of a novel procedure called ADS that can bridge this gap. **Methods:** Results from 18,464 ADS procedures were retrospectively analyzed for feasibility, safety and comfort. **Results:** 96.8% of ADS procedures were successfully completed without complication, confirming the efficacy of the procedure. **Conclusions:** ADS is a single procedure for the assessment of physiological functions of the upper aerodigestive tract, including swallow, voice, respiration, and cough while also screening for impaired gastric retention and obstructive sleep apnea, which may pose additional urgent and emergent airway threats. ADS may improve health outcomes in underserved populations, e.g., those in a rural community, referred to by other health professionals due to complaints of aerodigestive symptoms and signs.

## 1. Introduction

Our experience in rural healthcare in the United States of America, including community hospitals, physician offices, outpatient clinics, skilled nursing facilities, and home health agencies, is that many of these rural healthcare settings do not have cost-effective and timely access to instrumental assessments for physiological disorders of the upper aerodigestive tract. To further confound the problem, simple symptoms do not imply simple disorders, and a single professional’s knowledge base unfortunately does not cover all physiological disorders of the aerodigestive tract (AT). Although there are clinical disciplines which overlap, evidence suggests no single clinical discipline professes dominance for the diagnosis and treatment of all physiological disorders of the AT.

These various overlapping medical, surgical, and therapeutic disciplines inherently approach the process of diagnosing physiological disorders of the AT from their unique perspectives. Therefore, any single diagnostician is likely to focus solely on the chief complaint(s) most relevant to their discipline, risking a myopic view which may fail to identify significant comorbidities and in turn may lead to an increased likelihood of misdiagnosis, missed diagnoses, costly inappropriate referrals to other professionals, increased morbidity, or even death while deferring the most appropriate treatment. Unfortunately, this reality exacerbates when patients with physiological disorders of the AT face limited access to aerodigestive professionals in rural settings. This persistent healthcare conundrum in rural settings resulted in the demand by rural physicians and speech-language pathologists (SLPs) to develop a single differential diagnostic instrumental assessment for the comprehensive examination of all physiological functions of the AT. This motivation forged our concept of the aerodigestoscopy (ADS) procedure. 

The ADS procedure is designed to simultaneously evaluate all aerodigestive dysfunctions in a single exam which include but are not limited to swallow disorders (dysphagia) [1,2,3,4], motor speech disorders (dysarthria and apraxia of speech) [5,6,7], voice disorders [8,9,10], resonance disorders [11,12], cough disorders [13,14,15], breathing disorders [13,16,17,18], obstructive sleep disorders [19,20], and regurgitation disorders (dysemesis, backflow, and reflux) [21,22,23]. The impaired neuromuscular function of one part of the AT can result in a series of physiological dysfunctions elsewhere. For example, while impaired glottic closure (e.g., from unilateral vocal fold paralysis) can result in impaired voicing, it may also adversely affect the mechanical function of the thoracic cage muscles. The subsequent physiological deficits of the latter can result in further physiological impairments of airway clearance, defecation, micturition, and parturition, which can be thought of as tertiary deficits [24]. Physiological disorders of the upper aerodigestive tract can in turn lead to dehydration, malnourishment, weakness of the extremities, and generalized weakness with secondary issues of altered biochemistry, neurochemistry, cardiac chemistry, etc. Psychosocial and societal consequences of physiological disorders of the aerodigestive tract can include the compromise of mental health with depression and/or anxiety and occupational consequences [25]. 

In both optometry and audiology, similar differential algorithm-driven diagnostic procedures (eye exam and audiogram) with specific subcomponents are shared between physician and non-physician specialists. Unfortunately, in the world of diagnosing physiological disorders of the AT, there is no shared unified algorithm between aerodigestive professionals. However, in medically complicated cases involving the diagnosis and treatment of physiological disorders of the AT, a shared differential diagnostic algorithm between aerodigestive professions can save time and resources in addition to preserving the hope of a given patient who is waiting for a definitive diagnosis and treatment. In addition, such a shared diagnostic algorithm may optimize professional-to-professional communication. 

The origin of the ADS procedure in 1989 resulted from the first author’s desire to create a procedure with robust diagnostic yield across the spectrum of physiological disorders of the aerodigestive tract. To accomplish this goal, the first author began by surveying the various clinical and instrumental technologies and approved standards for the evaluation of various physiological disorders of the AT at that time. As the list of procedures began to grow, the next question posed was which of these procedures were capable of being integrated into a complementary unified differential diagnostic algorithm as sub-components with software guidance to evaluate each key physiological function of the upper aerodigestive tract. This challenge required ADS software development to guide the holistic AT assessment in an efficient manner that facilitated a seamless, intuitive progressive movement through the administration of the procedure protocol. These activities included data collection, analysis, and evidence-based recommendations for further diagnostics, consultation by the most appropriate overlapping aerodigestive professional, and/or treatment(s). Our awareness of the clinical and economic patient needs as reported by the medical community through surveys and cost analysis cited in the paragraph that follows motivated our development of the ADS system as just described. 

While statistics pertaining to the collective incidence and prevalence of physiological disorders of the AT across the disciplines of therapy, nursing, medicine, and surgery are lacking, there are some reports giving insight to the incidence and prevalence of some disorders of the AT. Swallow disorders have a prevalence of 3% of the adult population, thus affecting more than 9 million people in the US and causing over 60,000 deaths per year in the stroke population [26,27]. However, prevalence in the elderly may be significantly higher, with a reported average of 15%, and up to 60% in nursing homes [28,29]. The economic burden of dysphagia has been estimated to amount to USD 7 billion added to annual healthcare costs [26]. The observed mortality rate may be in part due to delayed or inaccurate diagnosis that overlooks significant comorbidities, resulting from the use of segmental instead of comprehensive diagnostic procedures [29,30]. Less information exists regarding the incidence and prevalence of voice disorders and motor speech disorders. Dysphonia has been shown to affect nearly one third of all persons across the lifespan [31]. Motor speech disorders (MSDs) include both dysarthria and apraxia of speech; according to a recent study by the Speech Pathology Section of the Mayo Clinic, MSDs account for about one half of the primary communication disorder in people who present with neuropathology, whereas at the same clinic, MSDs represent the primary communication disorder in over one third of all persons with an acquired disease of any type [32]. Gastroesophageal reflux disease (GERD) in North America has a reported prevalence of 18.1% to 27.8% [33]. The prevalence of obstructive sleep apnea (OSA) in the United States of America has been reported in the range of 3 to 7% [34].

The purpose of this paper is to examine the feasibility, safety, and comfort of the ADS procedure, present reasons for referral, and highlight the procedure’s diagnostic yields. 

## 2. Materials and Methods

### 2.1. Data Collection

All ADS procedures conducted from 2011 to 2020 were reviewed retrospectively to ascertain the feasibility, the safety, and the comfort of the ADS procedure. To assess the feasibility of the ADS procedure, the data were reviewed in terms of how many procedures were conducted in a hospital setting and how many were conducted in a skilled nursing home setting. An average time per procedure was calculated. The safety of the ADS procedure was evaluated by reviewing the medical records for any untoward safety issues including medical emergencies that may have arisen as a result of the procedure. Additionally, the adverse effects of epistaxis, mild vasovagal response (specifically fainting), and anaphylaxis reactions (to gloves, endoscope materials, food/liquid items, etc.) were tallied by type to calculate the incidence of those occurrences when present. Incidences were reported as a percentage. The comfort of the ADS procedure was examined by reviewing the number of patients who were recommended to have a follow-up procedure with the actual number of those who agreed to receive a follow-up procedure. 

### 2.2. Category 1 Materials

Durable Medical Equipment:

- JedMed 58 cm Extended Length Nasopharyngoscope;
- JedMed M-Camera;
- JedMed Medical EDA audiovisual capture software;
- ADS Software v. 1.0 developed by Southeastern Biocommunication Associates, LLC;
- Laptop computer with Windows operating system;
- Sphygnomonometer;
- Stethoscope;
- Dual Pulse Oximeter–Capnographer;
- Micro I Diagnostic Spirometer for Peak Expiratory Flow measurement;
- Micro-Respiratory Pressure Meter for Maximum Inspiratory Pressure (MIP) and Maximum Expiratory Pressure (MEP);
- Calibrated Microphone

### 2.3. Category 2 Materials

- Perishables and single use supplies;
- Infection control supplies;
- Sample food and liquid consistencies, clothes protectors, napkins, towels, utensils, surgical lubricant, etc.

### 2.4. Procedure

Detailed descriptions of each step of the ADS workflow can be found in the Supplementary Materials as well as a video covering steps P5 through P17 of the ADS procedure (see Supplementary Materials). The following is a brief overview of the steps of the procedure. 

Steps in ADS procedure

P1—Pre-ADS History and Physical;
P2—Perceptual Analysis of Voice;
P3—Acoustic Analysis of Voice;
P4—Spectrographic Analysis of Voice;
P5—Initial Oral Exam;
P6—Anterior Nasal Exam;
P7—Nasopharyngeal Exam;
P8—Velopharyngeal Exam;
P9—Reflux Finding Score (RFS);
P10—Stroboscopic Analysis of Voice;
P11—Pharyngeal and Laryngeal Exam Under Constant Light prior to the initiation of P.O. trials;
P12—Transesophageal Passage and the Gastric Screen;
P13—Esophageal Exam;
P14—Pharyngeal and Laryngeal Exam with P.O. Trials Augmented with the Clinical Swallow Exam (CSE);
P15—Re-examination of Esophageal Physiology;
P16—Final Velopharyngeal Exam;
P17—Final Oral Exam;
P18—Post-ADS Physical Exam;
P19—Augmentative/Supplemental Procedures if Applicable;
P20—Initiation of Data Analysis;
P21—Completion of Infection Control;
P22—Documentation.

A video covering steps P5 through P17 of the ADS procedure has been added as Supplementary Materials (or available under https://drive.google.com/drive/folders/1-JFzNGcfcBiOhA-1p4vjKR64g5dASb5R [accessed on 2 December 2024]). 

### 2.5. Patient and Public Involvement (PPI) Statement

While patients and public were not involved in the design of the procedure, it was developed with the benefit of the patients and the public in mind to expedite the amount of time from the identification of symptoms and signs to diagnosis and appropriate evidence-based treatment recommendations, which can represent a gap in care particularly in rural healthcare settings.

## 3. Results

**Figure 1:**
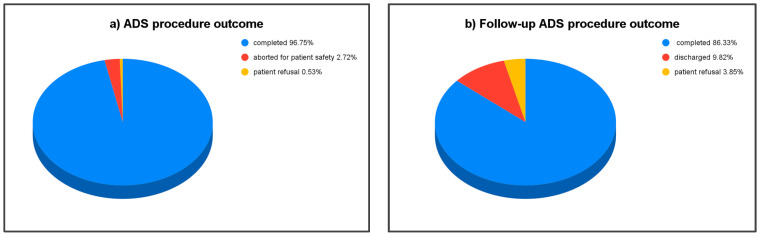
ADS procedure outcome. (**a**) Of a total of 18,464 ADS procedures that were initiated, 96.75% were completed (blue), 2.72% (red) were aborted for patient safety reasons, while 0.53% (yellow) were refused by the patients. (**b**) Of the recommended follow-up procedures, 86.33% (blue) were completed, 9.82% (red) were not completed due to patient discharge, and 3.85% (yellow) were refused by patients.

Patient records collected between 2011 and 2021 were assessed for ADS procedure completion. A total of 18,464 ADS procedures were ordered on adults aged 18 to 109 by the referring physicians between 1 January 2011 and 31 December 2021. Of these, 17,881 ADS procedures were completed, while 487 procedures (2.72%) were deemed as aborted procedures, which meant the procedure could not be performed as those patients presented as being outside of the vital sign parameters utilized for ensuring patient safety and/or presented with acute signs or symptoms of medical instability or distress. In addition, another 96 (0.53%) procedures were aborted due to patient refusal to permit the transnasal passage of the endoscope before or during endoscopy (Figure 1a). For patients unable to complete the procedure, a referral was made back to the ordering physician for further medical evaluation. 

Of the 17,881 completed, 7563 were recommended as follow-up ADS procedures after completing treatment recommendations. Of these, 6529 (86.33%) follow-up studies were completed. Of the 1034 patients who did not comply with the follow-up procedure, 291 (3.85% of total) did not comply due to report of fear of experiencing his/her pain or discomfort felt during the initial ADS procedure, whereas the remaining 743 (9.82% of total) patients did not have the recommended follow-up due to being discharged from his/her facility. This renders 3.85% of the patients who were recommended for the follow-up ADS as not complying due to what may be termed as intolerable pain/discomfort. The most common complaints made by this latter group of patients who refused were tickling of the nose, sneezing, coughing, pressure or pain in the nose. 

Patients were referred for the ADS procedure from physicians, physician assistants, nurse practitioners, nurses, and therapists practicing mostly in rural hospitals, outpatient clinics, and nursing homes across the states of Alabama and Mississippi. The reasons for referrals from physicians for the ADS are summarized Table 1.

**Figure 2:**
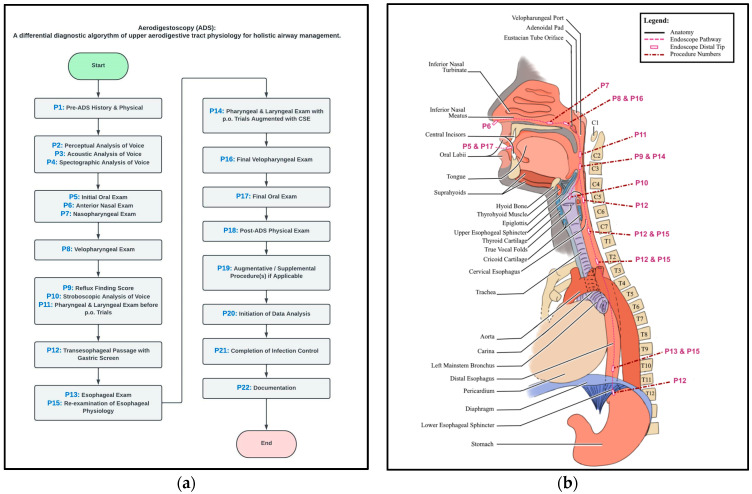
ADS pathway and anatomical site. Workflow of the ADS procedure (**a**) and anatomical site of each step (**b**). Detailed descriptions of each step as well as a video covering steps P5 to P17 are available in Supplementary Materials.

All patients who underwent the ADS procedure as outlined in Figure 2 were determined to be medically stable by their physicians and were within the ADS vital sign parameters reported in Table 2.

Patients who were eligible to undergo ADS were evaluated in accordance with the ADS workflow. 

### Adverse Effects

The pre and post vital signs were reviewed by the physician ordering the procedure as part of the review of the ADS report to ensure no other medical emergency issue(s) had arisen as a result of the procedure. Additionally, the adverse effects of epistaxis, mild vasovagal response (specifically fainting), and anaphylaxis reactions (to gloves, endoscope materials, food/liquid items, etc.) were tallied by type to calculate the incidence of those occurrences when present. 

For the 17,881 ADS procedures completed, no emergency medical personnel were required to manage the occasional adverse effect of mild epistaxis in 109 patients (0.60%). There were no instances of mild vasovagal response (fainting), life-threatening laryngospasm, or anaphylaxis. The risk of anaphylaxis was mitigated, as no topical anesthesia was used during endoscopy. Furthermore, patient complaints of discomfort (e.g., pressure in the nose, fear of gagging, and actual gagging) were rare.

## 4. Discussion

Here, we have described a novel and demonstrably safe endoscopic procedure for the differential diagnosis of primary and secondary physiological deficits of the entire aerodigestive tract from the nasal and oral cavities to the stomach. The ADS procedure is guided by an algorithm for the examination of the nasal cavity, oral cavity, pharynx, larynx, trachea, and esophagus, as well as a gastric screen, and it allows for physiological diagnosis of the upper aerodigestive tract while identifying comorbid airway threats from both above and below the level of the larynx. It enables the medical team to employ comprehensive airway management to examine a holistic array of treatment options for physiological disorders of the upper aerodigestive tract as guided by the chief complaint. Furthermore, ADS aims to optimize patients for healing and the rehabilitation process through the identification and management of airway threats, which at times can also result in an impaired ability to maintain nutrition and hydration. The ADS procedure is unique in that it includes both an esophageal exam and a gastric screen component to identify signs consistent with abnormal gastroesophageal function which, depending upon the underlying etiology, may present a primary or comorbid airway threat. 

ADS empowers the diagnostician to perform a comprehensive assessment of all physiological functions of the aerodigestive tract including but not limited to swallow impairments. The robust diagnostic yield of ADS is usually obtainable regardless of a given patient’s level of alertness, cognitive communication status, or seating and positioning limitations. ADS is cost-effective, as the initial instrumental assessment of aerodigestive physiological functions especially in rural skilled nursing facilities, outpatient clinics, physician offices, and community hospitals where fluoroscopy, endoscopy, stroboscopy, high-resolution manometry, and other advanced instrumentation are often not easily available. The ADS procedure with use of the ADS software typically requires one hour of a diagnostician’s time including set-up, chart review, actual endoscopy time, data analysis, and writing of the report. 

Today, videofluoroscopy with the traditional Modified Barium Swallow (MBS) [35,36] and utilizing the standardized MBSImp [37] are considered the “gold standard” for evaluating swallow physiology. However, the MBS is limited in its current form for screening for GI dysfunction, which at times may be the primary or significant comorbid issue presenting the greatest threat to the airway. For example, a recent study found that the coexistence of vomiting and dysphagia can indicate impaired gastric emptying [38], which can result in gastroesophageal bolus backflow with subsequent tracheal aspiration especially if there is a comorbid glottic closure impairment and/or laryngeal sensation impairment. It is not possible to reliably detect this with the MBS or the MBSImp. Another limitation is that the MBS is unable to provide clearance for the initiation of true vocal fold exercises which may be warranted when impaired glottic closure is the primary reason for the aspiration of food, liquid, and oral medications into the trachea. As the MBS is not well suited for ruling out a primary or comorbid GI dysfunction, clinicians using fluoroscopy alone are at risk for misinterpreting the difference between reflux, backflow, and emesis. One other limitation of the MBS is that it is not well suited for identifying comorbid impaired physiologies of the upper aerodigestive tract, which may exacerbate a given patient’s dysphagia and/or pose additional threats to airway safety (cough disorders, breathing disorders, reflux disorders, etc.).

Another standard for the assessment of swallow physiology is the fiberoptic endoscopic evaluation of swallowing (FEES) [39,40]. FEES is well equipped as a follow-up procedure for the further evaluation of pharyngeal swallow physiology and/or re-evaluation of pharyngeal swallow physiology after an MBS has been performed. In addition, in healthcare settings where fluoroscopy is unavailable, the FEES procedure administered in conjunction with a clinical swallow exam is well suited for evaluating both oral and pharyngeal swallow physiology as well as their inter-relationship. However, FEES alone, even when administered with a clinical swallow evaluation, is inadequate for the detection of comorbid esophageal dysphagia and gastroesophageal dysphagia. Without a stroboscopic component, it is also ill equipped for clearing a patient for true vocal fold (TVF) adduction exercises when impaired glottic closure is the primary or comorbid cause of aspiration for a given patient. Although ADS is not intended to replace the MBS, the MBSImp, or FEES, it has been our experience that the performance of the ADS procedure with its software containing guidance for interpreting the differential diagnostic algorithm has proved successful in identifying reasons why various patient types did not progress with the diagnostic yield of the MBSImp alone, FEES alone, or even cases when both the MBSImp and FEES were utilized in conjunction. 

Regardless of the chief complaint pertaining to the aerodigestive tract, the holistic approach provided by the ADS procedure for a complete exam of aerodigestive physiology can be utilized to minimize the risk of missing clinically significant comorbidities. The rationale here is similar to that employed in comprehensive differential diagnostic exams utilized in clinical vision science and clinical hearing science. For if each piece of a complex biomechanical system is not carefully examined, then there may be risks for less favorable clinical outcomes especially when all comorbid issues in a given system are not identified early in the diagnostic process. The clear, timely delineation of primary and comorbid physiological deficits of the upper aerodigestive tract is desirable when considering the selection of the most cost-effective holistic treatment(s) particularly given the existing evidence pertaining to cross-over treatment effects [24,41,42,43,44,45]. Such an approach to the identification and management of aerodigestive issues may be considered a superior philosophy of comprehensive airway management across populations. In terms of airway management, there is a sense of urgency for the need for the timely identification of comorbid aerodigestive disorders. This was highlighted by the advent of the difficult airway response team (DART) as well as the more recent call for speech-language pathologists to become members of DART teams [46,47]. This is exacerbated in the United States of America by progressively diminished reimbursement with Medicare-dependent as well as privately insured populations, as the number of elderly patients continues to rise each year. Another area of increased utilization in the face of reduced reimbursement is occurring with the increased incidence of late-stage head and neck cancers in the United States of America [48]. The complexity of diagnosing aerodigestive disorders has also recently been highlighted by the proposal of a multisystem swallowing framework which acknowledges the inter-relational support of various physiological systems potentially impaired during post-COVID-19 [49]. 

The 17,881 patients reported here tolerated this procedure involving unanesthetized transnasal passage of the flexible fiberoptic endoscope to just inside of the stomach without significant complications and minimal discomfort. Patient reports of discomfort included feeling pressure in the nose, elicitation of a cough and/or sneeze, and occasional elicitation of a gag reflex. However, when patients were instructed to concentrate on breathing normally through the nose, they reported a reduction in the sensation of nasal pressure. In addition, for patients who gagged, once cued to breathe in and out of the nose, they reported increased tolerance without gagging. The overall tolerance of the transnasal passage for the ADS procedure appears to be commensurate with other studies examining patient tolerance and comfort levels. Some studies found no statistically significant difference in comfort levels between subjects who received a local anesthesia and subjects who received a placebo for transnasal laryngeal examination procedures such as the FEES procedure [50,51]. Other studies have found transnasal pharyngoesophageal procedures including transnasal esophagoscopy exams and pharyngoesophageal high-resolution manometry to be feasible, safe, and well tolerated by patients [52,53].

The ADS algorithmic protocol has been shown to be comprehensive, as it examines the key physiological functions of the entire upper aerodigestive tract most relevant to a given patient’s chief complaint. The protocol is also flexible as it allows the diagnostician to consider the effect(s) of comorbid aerodigestive deficits upon the primary dysphysiological diagnosis of concern. ADS is distinct from other instrumental assessments of physiological functions of the aerodigestive tract as it empowers the diagnostician to identify primary and comorbid physiological impairments from the mouth and nose to the stomach; consider their potential adverse synergistic effects; and develop holistic differential diagnostic statements as well as comprehensive treatment recommendations. 

The philosophy of comprehensive airway management at the core of the ADS procedure can be described as follows: (1) to definitively rule out airway threats from above and from below the larynx; (2) to delineate the nature, extent, and severity of disordered physiology of the upper aerodigestive tract; and (3) to assess stimulability for potential evidence-based direct and indirect interventions. For the purposes of this paper, stimulability refers to signs consistent with a favorable prognosis for a specific therapeutic intervention. Indirect interventions include but are not limited to food, liquid, and medication consistency modifications, compensatory strategies (e.g., limited bolus size, cyclic ingestion, slow rate, multiple swallows, etc.), posture modifications (e.g., chin tuck, head rotation, etc.), and maneuvers (e.g., effortful swallow, supraglottic swallow, etc.). Direct interventions include but are not limited to evidence-based therapy interventions (e.g., Lee Silverman Voice Therapy, PhoRTE therapy, McNeil Dysphagia Therapy Program, combined respiratory muscle training, etc.). In addition to assessing stimulability for specific therapeutic interventions during the rehabilitation portion of the ADS procedure, modality trials can also be performed as part of the ADS procedure (e.g., neuromuscular electrical stimulation [NMES] mapping, surface electromyography [sEMG] mapping, etc.) as well as manual therapy trials (e.g., myofascial release, etc.). Lastly, part of the ADS algorithm is designed to identify when there is a need for physician consultation to evaluate and rule out a specific medical etiology as well as physician consultation to evaluate for a specific pharmaceutical intervention. In addition, components are built into the algorithm to identify symptoms and signs warranting a specialty physician or surgeon consultation for further evaluation. Although a detailed analysis of downstream referrals generated by the ADS is beyond the scope of this paper, anecdotal observations of the endoscopists who performed the ADS procedures in this study suggest referrals for further diagnostic work-up, care of urgent and emergent airway issues, and treatment procedures including but not limited to gastroenterologists, otolaryngologists, pulmonologists, radiologists, neurologists, neurosurgeons, head and neck surgeons, and gastrointestinal surgeons as well as speech, physical, occupational, and respiratory therapists. 

The ADS procedure also affords flexibility in terms of reducing healthcare disparities currently existing between urban healthcare and rural healthcare environments, as the latter often do not have immediate access to the armamentarium of procedures readily available in urban areas. The ADS procedure has been successfully deployed across the continuum of rural healthcare settings including community hospitals, outpatient clinics, physician offices, skilled nursing facilities, and home health. Lessons we have learned suggest that ADS may also be a cost and time-effective diagnostic platform for physiologically based aerodigestive disorders in urban healthcare settings.

The implementation of novel procedures can sometimes be delayed by clinical inertia and reluctance to change [54]. It is interesting to note here one of the seminal publications for the development of the modified barium swallow (MBS) by Dr. Jerilyn Logemann was published in 1967 [55], and yet the MBS is still not readily available for many residing in rural areas in the USA. Perhaps this reflects the difficulty in updating the human fund of knowledge. Given the potential impact on morbidity and mortality from aerodigestive disorders, we urge clinicians to accept the challenge to integrate this procedure into their diagnostic routine. The reason no single procedure for examining all of the cardinal physiological functions of the upper aerodigestive tract has been reported may reflect upon the upper aerodigestive tract not being considered as a separate and distinct body system in the classical sense (e.g., cardiovascular system, pulmonary system, etc.). Regardless, due to its various influences upon the integrity of airway safety, it warrants being considered as an interconnected synergized system for enabling multiple bodily functions while maintaining comprehensive airway regulation.

## 5. Conclusions

Aerodigestoscopy (ADS) is a feasible, safe, and reasonably comfortable medically minimally invasive endoscopic differential diagnostic procedure for physiological disorders of the upper aerodigestive tract. ADS offers a solution to healthcare disparities by providing a robust diagnostic option where access to specialized care is limited. Healthcare providers and institutions should prioritize the integration of ADS into rural and underserved healthcare systems to bridge the diagnostic gap. ADS also allows for the rapid identification of comorbid airway threats. Additional research is warranted to further investigate the cost-effectiveness, utility, and the diagnostic yield of ADS against the current standard of diagnostic care for the evaluation of aerodigestive physiological deficits.

## Figures and Tables

**Table 1:** Reasons for ADS referral(s) by chief complaint or clinical rationale.

Reasons for ADS Referral(s)	
**Swallow**	Difficulty swallowing Choking/coughing/strangling when eating and/or drinking Globus Sensation of solid food sticking in throat or in chest Pain with swallowing Unfavorable response to swallow therapy Nasal regurgitation of liquids, foods, and/or medication Loss of consciousness when swallowing Requirement of Heimlich maneuver when eating/drinking Drooling Tearing of eyes Wet vocal quality
**Voice**	Sudden onset of voice changes especially if persistent Gradual onset of voice changes Unfavorable response to voice therapy Unfavorable response to respiratory therapy especially with failed ventilator weaning attempts and/or failed endotracheal tube extubation attempts Suspicion for reduced laryngeal and/or tracheal airway patency Inability to tolerate one-way tracheostomy speaking valve
**Resonance**	Onset of hypernasality Onset of hyponasality Cul de sac resonance ℅ nasal emissions
**Cough**	Persistent cough Weakened volitional and/or spontaneous cough Cough of unknown origin Stridor Inability to produce a productive cough
**Respiration**	Difficulty breathing during or after meals Difficulty breathing in presence of negative chest X-Ray Intolerance of one-way tracheostomy speaking valves with tracheostomy patients Assess readiness for extubation of tracheostomy tubes Awakening from sleep coughing/choking
**Regurgitation**	Late-prandial or postprandial vomiting Suspicion of reflux H/o of persistent and/or worsening dysphagia
**Gastrointestinal**	Placement of transnasally passed gastric decompression tubes Placement or placement check of transnasally or transorally placed feeding tubes Weight loss Premature satiety Frequent constipation and/or diarrhea Absence of bowel sounds
**Other**	Fever of unknown origin Sepsis of unknown origin Biofeedback therapy session Follow up to assess progress with pharmaceutical, surgical and/or therapy interventions Frequently waking from sleep coughing/choking/strangling

**Table 2:** Recommended vital sign parameters that should be assessed before and after each ADS procedure. Patients outside of these parameters should only be considered under further physician consultation.

Vital Sign Parameters		
	Minimum Value	Maximum Value
Systolic blood pressure	86 mm Hg	180 mm Hg
Diastolic blood pressure	34 mm Hg	120 mm Hg
Pulse (bpm)	40 beats per minute	120 beats per minute
Respiratory rate	10 breaths per minute	40 breaths per minute

## Data Availability

Original raw data available upon request.

## Supplementary Materials

The following supporting information can be downloaded at https://www.mdpi.com/article/10.3390/jcm13247578/s1, Table S1: ADS Procedure Protocol; Table S2: Esophageal Dysphagia: Helpful terms and concepts; Table S3: Regurgitation Disorders: Helpful terms and concepts; Video S1: ADS Procedure; Video S1: ADS Procedure.
