# Supplementary material for: Aerodigestoscopy (ADS): The Feasibility, Safety, and Comfort of a Comprehensive Procedure for the Evaluation of Physiological Disorders of the Aerodigestive Tract
Source: J Clin Med. 2024 Dec 13;13(24):7578. doi: 10.3390/jcm13247578 (PMC11727860; doi:10.3390/jcm13247578)
Supplement: Supplementary file 1 [file jcm-13-07578-s001.zip › jcm-3304934-supplementary.pdf]

Supplementary material to

Aerodigestoscopy (ADS): The feasibility, safety, and comfort of a comprehensive procedure for the evaluation of physiological disorders of the aerodigestive tract

Table S1: ADS Procedure Protocol

Step in ADS procedure	Description
P1 - Pre-ADS History & Physical	Set up of the ADS equipment and the food stimulus items are performed according to infection control protocol(s). The ADS software guides the clinician through the collection of some data prior to transnasal passage of the scope, so the clinician can be aware of the conditions of other aerodigestive functions before performing the endoscopic exam. First, the patient’s medical history is reviewed, including the reason for the referral for ADS, with particular emphasis on any diagnosis related to the AT. A physical exam is then conducted, including the following components: oromotor and facial exam (focusing on both morphology and cranial nerve function), neck exam and thoracic exam, otoscopy, initial vital signs (blood pressure, pulse, respiratory rate, and indirect oxygen saturation), and pulmonary function tests (PFTs) as indicated. Also, the current route for nutrition, hydration, and medication (e.g. oral intake, nasogastric tube, PEG tube, etc.) as well as any oral intake restrictions in terms of food, liquid, and medication consistencies are noted. Additionally, the STOP-BANG can be administered to screen for OSA [34]. In case of the presence of a head and/or neck tumor, the TNM staging system data is entered as appropriate when available [35].
P2 - Perceptual Analysis of Voice	A perceptual analysis of vocal quality is performed, using the 4-point GRBAS scale (rating overall vocal severity, roughness, breathiness, asthenia, and

	strain)[36], while the ADS software records the audio sample. Additional specific perceptual descriptors (e.g. diplophonia, glottal fry, pItch breaks, tremor, etc) can also be reported here to enhance the perceptual analysis of voice.
P3 - Acoustic Analysis of Voice	An acoustic analysis of voice is performed. Measurements include: fundamental frequency in Hz, vocal intensity in dB SPL (along with maximum phonation time (MPT) and cepstral peak prominence (CPP). All measurements are made with both a sustained vowel and a short connected speech sample. All measurements are taken in a quiet exam room while using a calibrated microphone placed at a fixed distance from the patient's mouth using high-definition audio recording via the ADS software [37].
P4 - Spectrographic Analysis of Voice	The spectrographic analysis of voice is plotted by the ADS software for visual analysis in the corresponding area of the ADS report for both voice samples taken during P3 [37].
P5 - Initial Oral Exam	The endoscope is utilized to examine the oral cavity as it appears prior to the initiation of p.o. trials, and any abnormal findings are documented.<br>NOTE: A supplemental ADS flowchart as well as an anatomical depiction of the general position of the distal tip of the endoscope for each component of the ADS entitled "ADS Pathway" are found in the supplemental materials to enhance the description of the ADS procedure for P5 through P17.
P6 - Anterior Nasal Exam	The endoscope is gently introduced to each naris to examine each side of the nasal cavity to determine any anatomical differences which may pose challenges for safe passage of the flexible fiberoptic

	endoscope. Additionally, relevant signs consistent with disorders, diseases, or physiological dysfunction are noted. The use of nasal saline, surgical lubricant, and oxymetazoline HCl, can be considered as needed.
P7 - Nasopharyngeal Exam	As the scope is passed to the nasopharynx, imaging of the orifices of the Eustachian tubes as well as the adenoidal pad is performed, noting any abnormalities.
P8 - Velopharyngeal Exam	The flexible fiberoptic endoscope is then utilized to complete an examination of velopharyngeal function. This subcomponent of the ADS procedure contains speech tasks to be performed to rule out velopharyngeal insufficiency (VPI), which can adversely impact nasal resonance, and swallow tasks to rule out velopharyngeal dysfunction (VPD). VPD can result in foods, liquids, and/or oral medications entering the posterior nasal cavity as well as result in a decrease of pharyngeal pressure and subsequent esophageal pressure for functional bolus passage. Signs of erythema, edema, and drainage from the adenoidal pad consistent with seasonal allergy(ies) are noted. In addition, any erythema, edema, or cobblestoning of the mucosa ascending from the pharynx into the inferior nasopharynx consistent with exposure to gastric acids are noted.
P9 - Reflux Finding Score (RFS)	The endoscope is now advanced further into the pharynx to a position superior to the laryngeal vestibule and one or more images are taken to be analyzed by using the Reflux Finding Score (RFS) for the detection of signs characteristic of laryngopharyngeal reflux (LPR) [23].
P10 - Stroboscopic Analysis of Voice	For the stroboscopic analysis of voice, the ADS procedure utilizes the Voice-Vibratory

	Assessment with Laryngeal Imaging (VALI) [38]. The VALI provides a framework for comprehensive stroboscopic examination of the larynx. It is ideal to perform the stroboscopic component of the ADS procedure prior to exposing the larynx and pharynx to food and liquid materials which may hinder full visualization of the glottis.
P11 - Pharyngeal & Laryngeal Exam Under Constant Light prior to the initiation of p.o. trials	For this portion of the procedure, a gross assessment of the pharyngeal and laryngeal musculature is performed without p.o. trials. This can help to modify or develop new clinical hypotheses before the p.o. trials portion of the ADS procedure. This section begins with a physical screening for anatomical signs which may place an individual at risk for a pharyngeal swallow disorder, a voice disorder, OSA, etc.. In addition, it includes gross evaluation of the movement of some key structures of the larynx and pharynx (eg vocal fold adduction, abduction, pharyngeal contraction and shortening, etc).
P12 - Transesophageal Passage and the Gastric Screen	Complete assessment of physiological functions of the upper aerodigestive tract requires an esophageal physiology exam as well as a gastric screening procedure. The gastric screen portion of this algorithm is based upon a four hour gastric emptying procedure utilized in nuclear medicine [39,40]. The gastric screen required the patient to be held *nil per os* (NPO) meaning nothing by mouth except for required oral medications, small volume medically necessary snack, or water, for at least 4 hours before the initiation of the ADS procedure. When possible, the Gastric Screen portion of the ADS is performed prior to the initiation of p.o. Trials. Prior to the passage of the endoscope through the cervical esophagus, a given patient's non-

	oral versus oral status is considered. Once the endoscope is inside of the cervical esophagus, the clinician visualizes the appearance of the mucosa of the esophagus while advancing inferiorly to the lower esophageal sphincter (LES). The gastric screen portion is considered to be passed when the patient’s stomach is less than half full and failed when the patient’s stomach is found to be greater than half full. When a patient fails the gastric screen, then the clinician documents the finding of signs characteristic of abnormal gastric retention versus impaired gastric emptying. An abnormal physiological diagnosis of gastroesophageal dysphagia is made when the patient fails the gastric screen. An abdoöinal X-ray versus CT of the abdomen is recommended. Depending on the findings, a GI physician consultation is recommended.
P13 - Esophageal Exam	NOTE: A table entitled “Esophageal Dysphagia: Helpful terms and concepts” as well as a table entitled “Regurgitation Disorders: Helpful terms and concepts” can be found in the supplemental material, both of which contain definitions to distinguish between these dysphysiological diagnostic terms as they relate to ADS esophageal findings.<br>Once the gastric screen is completed, then the clinician can elect to start initial p.o. trials with the distal tip of the endoscope positioned in the pharynx versus positioned in the distal esophagus. Which portion of the aerodigestive tract to assess first with p.o. trials largely depends upon the data collected at step P1 when completing the Pre-ADS history and physical. Assuming the clinician elects to initiate p.o. trials in the esophagus, the endoscope is typically withdrawn to a level approximately 2 to 4 cm above the LES. Any anatomical

	irregularities of the LES and distal esophagus are described. It is desirable to screen for achalasia of the LES prior to the administration of any solid consistency foods. To screen for signs of achalasia of the LES, liquid trials may be initiated in 5 to 10 cc bolus sizes with the given patient's current tolerated liquid consistency. In the event the total volume of the liquid consistency approaches 2 to 3 ounces while remaining mostly above the LES for longer than 5 minutes, then achalasia of the LES is suspected. This endoscopic screening methodology for detecting achalasia at the level of the LES is predicated upon an upright timed barium esophagram procedure [41,42]. In this case, the clinician makes a dysphysiological diagnosis of neurogenic gastroesophageal dysphagia characterized by signs consistent with achalasia of the LES. Referral is made to gastroenterology for appropriate tests to definitively rule in or rule out it's presence (e.g. EGD with retroversion while inside the stomach to definitively rule out the presence of a mass in the fundus, high resolution manometry to definitively rule out failure of the LES to relax, etc.).<br>*Motility Screening*<br>After completing the screen for signs consistent with achalasia of the LES, the clinician notes the presence of any significant delay in the esophageal emptying of the liquid characteristic of primary and/or secondary dysmotility of the esophagus [43,44]. Any tertiary contractions are also noted. When the gastric screen and the LES achalasia screen are passed, any esophageal emptying differences related to impaired peristalsis would warrant a dysphysiological diagnosis of neurogenic esophageal dysphagia. To completely assess motility, the distal tip of

	the flexible fiberoptic endoscope is withdrawn to just inferior to the aortobronchial compression at which time some bolus trials of various liquid and food consistencies may be conducted to observe the onset of the primary peristaltic wave as well as the clearance effect of the secondary peristaltic wave. Further motility studies may be warranted with high resolution manometry depending upon severity. Once esophageal p.o. trials are completed, final screening for any signs consistent with reflux, candida, etc. can be performed.<br>*Prolonged Solid Bolus Fixation Screening*<br>Next, screening is performed to rule out esophageal narrowing with the use of a standardized bolus (e.g. 13 mm placebo pill, or solid food such as 13 mm diced peaches) [44]. In the event a focal prolonged solid bolus fixation greater than 30 seconds with the 13 mm solid bolus is found, then a physiological diagnosis of obstructive esophageal dysphagia is made while referring for a GI physician consultation for definitive medical workup (e.g. EGD with esophageal dilatation and/or biopsy, if indicated, to ascertain and treat the underlying medical etiology of the obstructive esophageal dysphagia).
P14 - Pharyngeal and Laryngeal Exam with P.O. Trials Augmented with the Clinical Swallow Exam (CSE)	The distal tip of the endoscope is now withdrawn up to the level of the pharynx, and the pharyngeal and laryngeal swallow physiology components of the procedure are performed. This consists of an integration of the Fiberoptic Endoscopic Evaluation of Swallowing (FEES) [45–47]; the Murray Secretion Scale [48]; the Yale Pharyngeal Residue Severity Scale (YPRS) [49,50]; and the Penetration-Aspiration Scale (PAS) [51]. The endoscopist should be aware of the potential impact of the oral

	and velopharyngeal physiology on pharyngeal swallow function, as well as the influence of comorbidities including but not limited to pharyngoesophageal, esophageal, and gastroesophageal dysphagia. In addition, the effects of comorbid voice disorders, cough disorders, respiratory muscle weakness, dyspneas, and regurgitation (reflux versus backflow versus emesis) upon pharyngeal swallow physiology and collective airway safety of a given patient's upper aerodigestive tract should be considered. If signs of pharyngeal and/or laryngeal lymphedema are found, then the Revised Patterson Edema Scale is scored [52]. If the patient has a tracheostomy tube, then a subglottic screen photo may be taken looking inferiorly from the posterior glottis to assess airway patency at the level between the glottis and the uppermost portion of the cannula. Furthermore, a lower trachea screening photo may be taken to assess lower airway patency as well as pulmonary toilet by passing the flexible fiberoptic endoscope via the cannula to the distal portion of the trach tube. It is important that clinicians use solid clinical reasoning when introducing p.o. trials during the ADS procedure. For example, if a patient has been strictly nothing by mouth and receiving all intake non-orally (e.g. PEG tube, PEJ tube, etc.), then after an initial visualization of the stomach and the esophagus some p.o. trials may be first initiated with examination at the level of the pharynx. This is to first rule out airway threats occurring immediately before, during, or immediately after the pharyngeal swallow reflex. Once this has been determined, then the clinician may re-introduce the endoscope into the esophagus to complete the esophageal component of the ADS procedure.

P15 - Re-examination of Esophageal Physiology	Although the gastric screen and the initial esophageal swallow components have been completed prior to initiation of the full pharyngeal-laryngeal exam, it is important to re-examine the stomach and the esophagus as the study progresses in order to differentiate which findings are primarily a result of abnormal pharyngeal and/or laryngeal physiology versus which findings are a primary versus a comorbid synergistic effect from abnormal esophageal and/or gastric findings.
P16 - Final Velopharyngeal Exam	Prior to removal of the endoscope from the nasal cavity, the velopharynx and nasal cavity are re-examined. As the endoscope is withdrawn, the clinician notes any signs of the color contrast material being expressed on the tissues within or above the velopharyngeal port, adenoidal pad, Eustachian tube orifices, nasal turbinates, etc. which may be additional evidence of velopharyngeal function being grossly impaired.
P17 - Final Oral Exam	Now a post-ADS oral exam is conducted and documented with a photo(s) for later data analysis of any oral swallow physiology dysfunction which may have occurred during the procedure. Additional visual clinical observations of oral stasis viewed by cues for the patient to open his/her mouth at intervals during the ADS procedure are also noted.
P18 - Post-ADS Physical Exam	Vital signs are taken again and documented after the procedure is completed. Any adverse effects of the ADS procedure (e.g. mild epistaxis) are reported along with any first aid that was administered. In the event that the patient tolerated the procedure well without any adverse effects, then a statement to this

	effect is documented into the ADS software for the clinical report.
P19 - Augmentative/Supplemental Procedures if Applicable	A re-introduction of the endoscope may be performed if needed for any augmentative/supplemental procedures (NMES mapping, sEMG mapping, therapeutic swallow maneuver trials after brief practice without scope in situ, myofascial release trials with follow-up imaging, FEEST, manometry, etc.).
P20 - Initiation of Data Analysis	As the audiovisual data is reviewed, specific photos as well as brief audiovisual samples of the salient findings are identified and saved to be posted into the ADS clinical report via the ADS software. When a given still photo or brief video file is posted into the storyboard portion of the ADS clinical report, the clinician has the opportunity to write a brief statement of what is salient in the relevant section of the storyboard portion of the report.
P21 - Completion of Infection Control	At this time, post-procedure infection control protocols are now executed in accordance with government regulations, professional organization guidance, manufacturers guidelines, and local facility protocols.
P22 - Documentation	Documentation of the completed ADS procedure is accomplished with the use of the ADS software. The ADS software generates a report including a set of digital worksheets used to enhance clinical interpretation of findings from each portion of the aerodigestive tract. The next component of the ADS report is a storyboard which is used to highlight any physiological abnormalities found with accompanying photos and videos to illustrate the interpretation(s) made. Furthermore, each section of the storyboard is used to present the results of

	any stimulability testing using direct or indirect interventions trialed during the ADS procedure. Lastly, the ADS report summarizes two sets of recommendations. The first set of recommendations are for the safest food, liquid, and oral medication consistencies based upon the ADS findings, as well as recommended methods of safe administration of oral intake (compensatory strategies, swallow maneuvers, positioning of the head, neck, and upper torso, etc). The second set of recommendations are the recommendations for specific evidence-based therapeutic intervention(s), modalities, and manual therapies, for which the patient appeared most stimulable during the procedure. In addition, any recommended specialist consults (e.g. GI, ENT, pulmonology, radiology, physical therapy, etc) are stated along with supporting rationale.<br>A video covering steps P5 through P17 of the ADS procedure has been added as supplemental material (or available under https://drive.google.com/drive/folders/1-JFzNGcfcBiOhA-1p4vjKR64g5dASb5R).

Table S2: Esophageal Dysphagia: Helpful terms and concepts.

Disorder	Definition
Pharyngoesophageal Dysphagia	Disordered swallow physiology in which normal pharyngeal bolus clearance fails to occur due to interruption of bolus passage from the hypopharynx into the cervical esophagus. NOTE: It is important to ensure impaired hypopharyngeal bolus emptying is not secondary to the presence of severe esophageal stasis.

Esophageal Dysphagia (Unspecified)	Disordered swallow physiology resulting from an unspecified impaired bolus flow through the esophagus.
Obstructive Esophageal Dysphagia	Disordered esophageal swallow physiology which involves a prolonged solid bolus fixation greater than or equal to 30 seconds with a 13 mm placebo tablet/pill and/or 13 mm diced/cubed peaches resulting from narrowing of the esophageal lumen at one or more specific points along its longitudinal axis.
Neurogenic Esophageal Dysphagia	Disordered esophageal swallow physiology which involves a deficit of the primary and/or secondary peristaltic waves resulting in delayed esophageal bolus clearance. This may be with or without tertiary esophageal contractions.
Pseudo-obstructive Esophageal Dysphagia	Disordered esophageal swallow physiology in which prolonged bolus fixation < 30 seconds in duration is found to be resulting from edema induced narrowing of the lumen typically of 1/3rd or more of the length of the esophagus.
Gastroesophageal Dysphagia	Impaired esophageal swallow physiology in which normal esophageal bolus clearance fails to occur due to the interruption of bolus passage at the Lower Esophageal Sphincter (LES). NOTE: It is important to ensure delayed esophageal emptying is not secondary to severe gastric retention/severely impaired gastric emptying.

Table S3: Regurgitation Disorders: Helpful terms and concepts.

Disorder	Definition
Regurgitation	A reversal of normal bolus flow in which foods, liquids, and/or oral medications swallowed have returned back to the level of the esophagus, pharynx, posterior oral cavity, or nose.

Reflux Disorders (GERD & LPR)	Is a specific regurgitation disorder in which excess stomach acid has been propelled up into the esophagus. In severe cases, the acid refluxate may be propelled up to the levels of the pharynx, larynx, posterior oral cavity, and posterior nasal cavity. NOTE: Acid reflux more commonly occurs when one is lying horizontal while asleep, also known as Nocturnal Reflux.
Backflow Disorders	A) Esophageal Backflow refers to the return of swallowed materials which did not empty into the stomach to superior points in the upper esophagus, pharynx, posterior oral cavity, and/or nasal cavity. NOTE: This is a sign consistent with obstruction at some level within the esophagus.
	B) Gastroesophageal Backflow refers to the return of swallowed materials which did not empty into the duodenum to superior points in the esophagus, pharynx, posterior oral cavity, and/or nasal cavity. NOTE: This is a sign consistent with obstruction at the level of the gastric outlet and/or below versus GI paresis versus paralysis.
Emesis Disorder (Dysemesis)	Impairment of vomiting in which the stomach contents are unable to be evacuated when required by the nervous system attempting to purge infectious agents (e.g. virus, bacteria, toxins, parasites, etc.) from the GI tract.

Video S1: ADS Procedure
https://drive.google.com/drive/folders/1-JFzNGcfcBiOhA-1p4vjKR64g5dASb5R
